# A mathematical model to predict network growth in *Physarum polycephalum* as a function of extracellular matrix viscosity, measured by a novel viscometer

**DOI:** 10.1098/rsif.2024.0720

**Published:** 2025-03-05

**Authors:** Philipp Rosina, Martin Grube

**Affiliations:** ^1^ Department of Biology, University of Graz, Graz, Austria

**Keywords:** extracellular matrix viscosity, fractal dimension, mathematical modelling, network growth, *Physarum polycephalum*, viscometer

## Abstract

*Physarum polycephalum* is a slime mould that forms complex networks, making it an ideal model organism for studying network formation and adaptation. We introduce a novel viscometer capable of accurately measuring extracellular slime matrix (ECM) viscosity in small biological samples, overcoming the limitations of conventional instruments. Using this device, we measured the relative kinematic viscosity and developed continuous models to predict network size over time as a function of ECM viscosity. Our results show that increased ECM viscosity, driven by higher salt (MgCl_2_·6H_2_O) concentrations, significantly slows network expansion but does not affect the final network complexity. Fractal dimension analysis revealed that network complexity converged to a similar value across all viscosity conditions during the equilibrium state. The models demonstrated strong predictive power, with a mean squared error below 0.4%, closely aligning with experimental data. These findings highlight the critical role of ECM viscosity in influencing network expansion while demonstrating that complexity remains stable across varying conditions. This study advances our understanding of the physical parameters shaping *P. polycephalum* networks and provides a foundation for exploring network dynamics in other adaptive systems. These insights offer new tools for research in biological systems where sample material is limited.

## 1. Introduction

*Physarum polycephalum* is a large, single-celled amoeboid organism renowned for forming intricate vein networks, enabling it to navigate its environment, solve problems and adapt to various stimuli. Its behaviours, including learning, memory and decision-making, have made it a valuable model for studying adaptive strategies in biological systems [1–3]. Notably, *P. polycephalum* has been observed to solve mazes by finding the shortest paths between multiple food sources. This behaviour mimics the Steiner minimum tree, wherein it creates optimized networks balancing efficiency and resilience [4–6]. These network-forming abilities are driven by cellular mechanisms, such as chemical oscillations and wave propagation, which enable the organism to navigate complex environments and optimize its resource distribution [7–9]. In addition to its problem-solving capabilities, *P. polycephalum* exhibits chemotactic responses that guide its movement towards attractants like amino acids, sugars and cyclic nucleotides while avoiding harmful substances [10–12]. Its transcriptomic plasticity and extensive alternative splicing contribute to its adaptability under changing environmental conditions [13,14]. The organism has also shown a remarkable ability to adapt its network structures in response to environmental pressures, efficiently shrinking or expanding its network to maintain functionality and resilience [15,16]. Furthermore, *P. polycephalum* interacts with light through a phytochrome-like photoreceptor, which regulates its sporulation and life cycle in response to environmental light conditions [17,18]. These unique characteristics of *P. polycephalum* have contributed to its establishment as a model organism for exploring fundamental principles of network optimization, resource allocation and bio-inspired algorithms, with applications in robotics, computational systems and adaptive biological networks [9,19,20]. Fractal geometry is useful for exploring these remarkable properties of *P. polycephalum*, particularly its complex network dynamics, providing insights into the structural complexity of such networks.

The concept of fractal dimension (FD) has been widely applied in the study of complex networks, offering insights into the filling ability and structural complexity of networks. In network systems, the FD serves as a critical indicator of how a network occupies space and adapts to changes, providing a measure of robustness under various conditions. Studies on different types of networks, from biological systems to urban infrastructure, have demonstrated a strong correlation between FD and network resilience. In natural systems, such as stream networks, FD have been used to investigate scaling properties, with typical values falling between 1.5 and 2, reflecting the balance between randomness and structure. Moreover, recent work has extended the concept of FD to graph theory, identifying how self-similarity and fractality in discrete networks like those of viral strains can reveal patterns of adaptation and evolution. These applications of FD are directly relevant to the structural analysis of *P. polycephalum* networks, where the FD quantifies the complexity of network structures and their ability to adapt to environmental constraints [21–23]. Understanding the complexity of *P. polycephalum* networks requires a deeper examination of the organism’s underlying biological properties, particularly its cytoplasmic dynamics and the extracellular matrix (ECM), which are key factors in network formation and adaptation.

The cytoplasmic properties of *P. polycephalum* have been extensively studied, revealing its role as a non-Newtonian viscoelastic fluid with anisotropic characteristics [24–26]. This dynamic behaviour, driven by rhythmic cytoplasmic streaming regulated by calcium ion signals, is critical for its locomotion and nutrient transport [27]. The secretion of extracellular slime, a prominent component of the ECM in *P. polycephalum*, plays a pivotal role in pseudopodial extensions and adhesion to substrates during movement [27,28]. The ECM in *P. polycephalum* comprises glycoproteins and secreted slime vesicles, forming a matrix that supports its structural organization and environmental adaptability [27]. Notably, the viscosity of the ECM can be modulated by introducing magnesium chloride (MgCl_2_), which increases the viscosity by influencing the ionic environment and interaction among ECM components. This property has been exploited in this study to systematically investigate the impact of ECM viscosity on network dynamics, highlighting MgCl_2_ as a critical factor in manipulating the extracellular milieu [29]. Furthermore, plasmalemma invaginations, which vary based on environmental conditions, such as nutrient availability, enhance the surface area for nutrient uptake and secretion, adapting the organism to fluctuating environments [30]. These dynamic and adaptive properties underline the ECM’s essential role in facilitating the unique behaviours of *P. polycephalum*, such as optimizing foraging strategies and network formation [18].

The ECM’s role extends beyond slime moulds to critical functions in human pathologies, particularly cancer. Altered ECM stiffness, driven by increased collagen density and cross-linking, is a hallmark of solid tumours, contributing to mechanotransduction and tumour progression [31–33]. Cancer cells exploit ECM remodelling to enhance invasion, metastasis and immune evasion by modulating stiffness and biochemical signals [34,35].

These changes hinder immune infiltration and therapeutic efficacy, emphasizing the importance of understanding ECM dynamics in the tumour microenvironment [36]. Insights from studies on ECM elasticity and structural organization have informed cancer therapies aimed at targeting ECM stiffness to improve drug delivery and immunotherapy outcomes [33,34]. By bridging ECM studies in *P. polycephalum* with cancer research, this work highlights the viscometer’s potential to provide a deeper understanding of ECM properties in diverse biological contexts.

Building on these previous studies, this research introduces a novel viscometer and mathematical models to investigate the relationship between ECM viscosity and network dynamics in *P. polycephalum*. The viscometer enables precise measurement of ECM viscosity in small biological samples, a critical advancement for limited sample size studies. The models developed in this study explore how viscosity influences network growth and structural complexity, providing new insights into the adaptive strategies employed by *P. polycephalum*. This research deepens our understanding of *P. polycephalum* and provides a foundation for exploring network optimization and adaptability in other biological and engineered systems.

## 2. Material and methods

**Figure 1.**
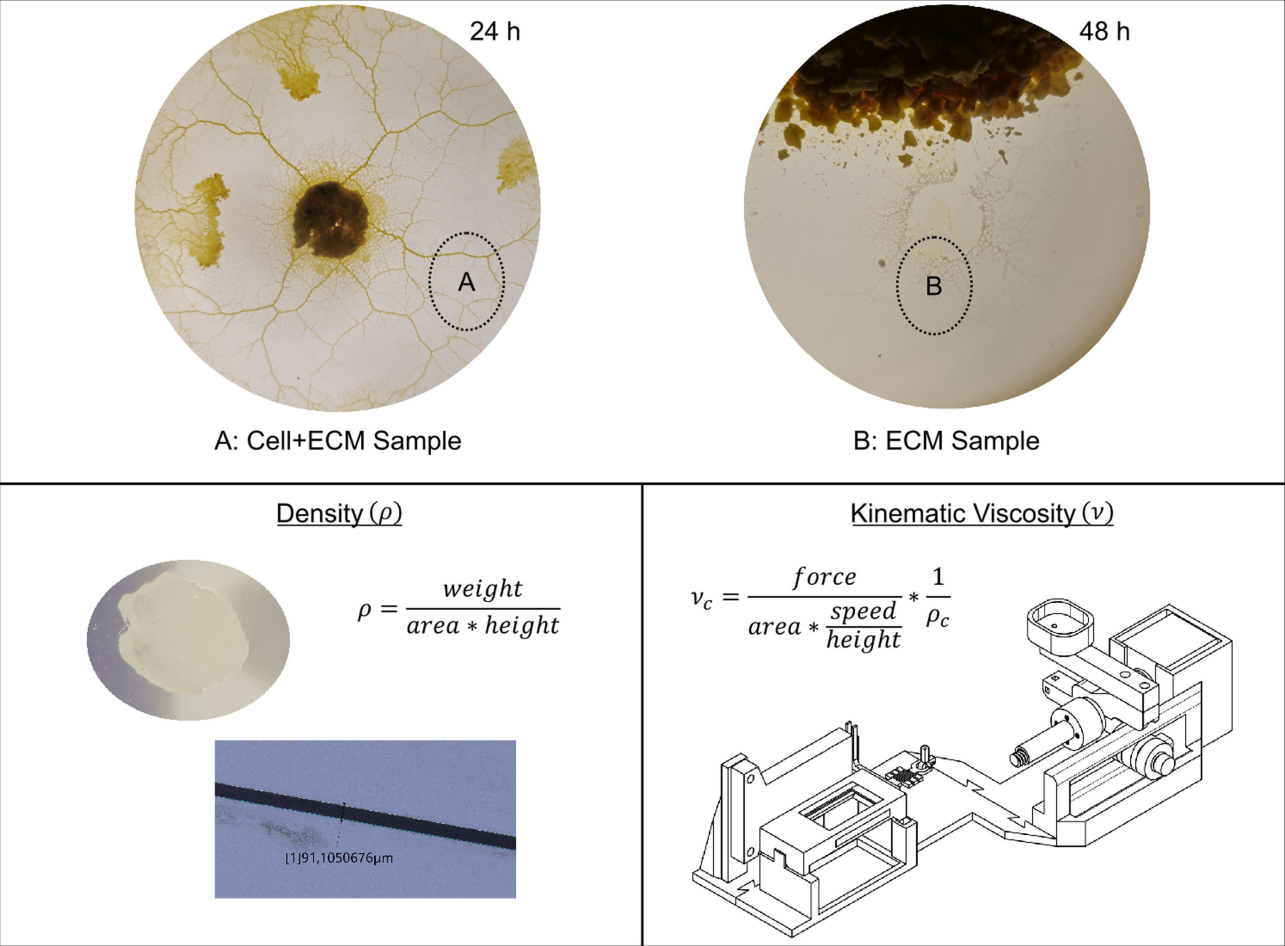
Overview of experimental procedures and techniques used in this study. *Physarum polycephalum* cultivated on agar plates with MgCl_2_·6H_2_O. Cell+ECM samples (A) were collected at the 24 h time point and ECM samples (B) were collected at the 48 h time point. Density measurement technique: this panel includes the equation used to calculate the density—the smaller images demonstrate how the area and height were measured to determine the volume of the samples. Kinematic viscosity measurement technique: this panel displays the equation for kinematic viscosity and an isometric view of the computer-aided design (CAD) model of the custom-engineered viscometer.

The experimental procedures and techniques used in this study are summarized in figure 1. *Physarum polycephalum* was cultivated on agar plates with MgCl_2_·6H_2_O and samples were collected at two distinct time points. Samples containing *P. polycephalum* and the ECM (Cell+ECM) (figure 1A) were collected at the 24 h time point, while samples containing only the ECM (figure 1B) were collected at the 48 h time point. Density measurements were conducted using a custom approach that involved calculating the sample volume based on area and height, as shown in the smaller images within the figure. Kinematic viscosity was determined using a custom-engineered viscometer, with the corresponding equation and an isometric view of the viscometer model displayed in the figure. These techniques allowed for precise quantification of the physical properties of the ECM and were critical to the development of the mathematical models presented in this study.

### 2.1. Cultivation and sample preparation

*Physarum polycephalum* (no. LU352) was cultivated in sterile Petri dishes (Sarstedt, 92 × 16 mm, no. 82.1473.001) containing 1.2% Agar–Agar (Roth, Agar–Agar Kobe I, no. 5210) with Milli-Q water (Milli-Q IQ7003, Q-Pod, Merck). MgCl_2_·6H_2_O (Roth, Magnesium chloride hexahydrate, no. 2189.1) was added to the standard agar mixture for the experiments at three different conditions: 10, 25 and 50 mM. The addition of MgCl_2_ alters the extracellular environment by modulating ionic interactions and increasing ECM viscosity, as previously demonstrated to influence cell adhesion and aggregation dynamics in cellular slime mould [29]. The slime mould was fed with extra-soft oat flakes (Kölln Flocken, Peter Kölln GmbH & Co. KGaA, Germany). In all cases, the agar mixture was autoclaved. Cultivation was performed under aseptic conditions to prevent microbial contamination. This approach ensured continuous exposure of the organism to MgCl_2_·6H_2_O, guaranteeing that the organism was influenced throughout the entire experiment to maximize the impact. Cultivation was carried out in the dark at a temperature of 22°C.

A 15 mm circular fragment of *P. polycephalum* was carefully transferred onto an agar plate, either supplemented with or without MgCl_2_·6H_2_O. The plate was monitored over 24 h to assess growth dynamics and the complexity of the resulting network. Following this incubation, the network was analysed to determine the density or viscosity of the slime mould samples. The original fragment was then removed and fresh oat flakes were positioned near the existing network to facilitate further ECM measurement. After an additional 24 h period, the entire slime mould was transferred to a new substrate of oat flakes, leaving behind an ECM residue on the plate. This allowed for the collection of pure ECM samples for subsequent density or viscosity analysis. All measurements were conducted immediately upon sample collection to minimize alterations, such as dehydration.

### 2.2. Density measurement techniques and data extension approach

The density of the ECM samples was determined by measuring both the volume and weight of the samples. Samples were collected using a clean spatula and transferred onto a microscope glass slide. The weight was measured in grams (later converted to kilograms) using a high-precision scale (Avantor/VWR, no. SM2285Di-ION-C). After weighing, another microscope glass slide was placed on top of the sample and the distance between the slides (representing the height of the sample) was measured in micrometres (later converted to metres) using a digital microscope (Keyence VHX 7000).

An image of the sample was captured with a smartphone (Samsung S24 Ultra) at 50 MP and 2.1 × magnification. A reference object of known size was included in the image to convert the pixel data to a metric scale. The area of the sample was measured using ImageJ (v. 1.53q), where the freehand selection tool was used to outline the sample and the pixel count was recorded. The pixel area was then converted into metric scale using the reference object.

With these variables, the density was calculated using equation (2.1). Multiple replicates were performed to ensure accuracy and precision.


(2.1) $$\rho =\frac{weight\left(kg\right)}{area\left(m^{2}\right)\cdot height\left(m\right)}.$$

**Figure 2.**
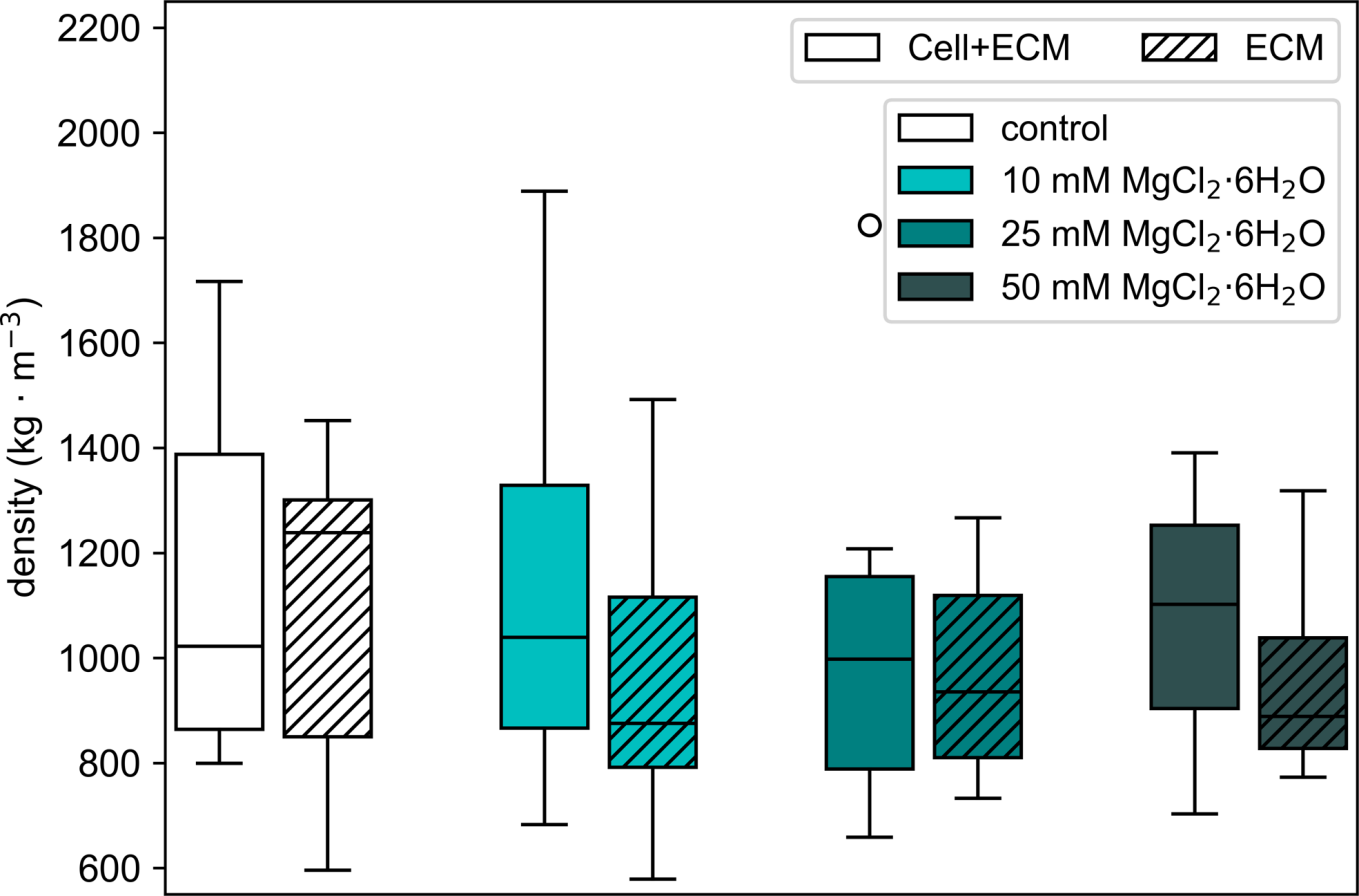
Density measurements of *P. polycephalum* samples at various concentrations of MgCl_2_·6H_2_O. The box plot shows the mean density of Cell-ECM and ECM samples for each condition: control (no additive), 10, 25 and 50 mM MgCl_2_·6H_2_O. Error bars represent the standard deviation of the measurements. The solid bars represent the Cell + ECM samples, while the hatched bars represent the ECM samples. Statistical tests were performed comparing Cell-ECM versus ECM and each condition versus control; no significant differences were detected in either comparison.

**Figure 3.**
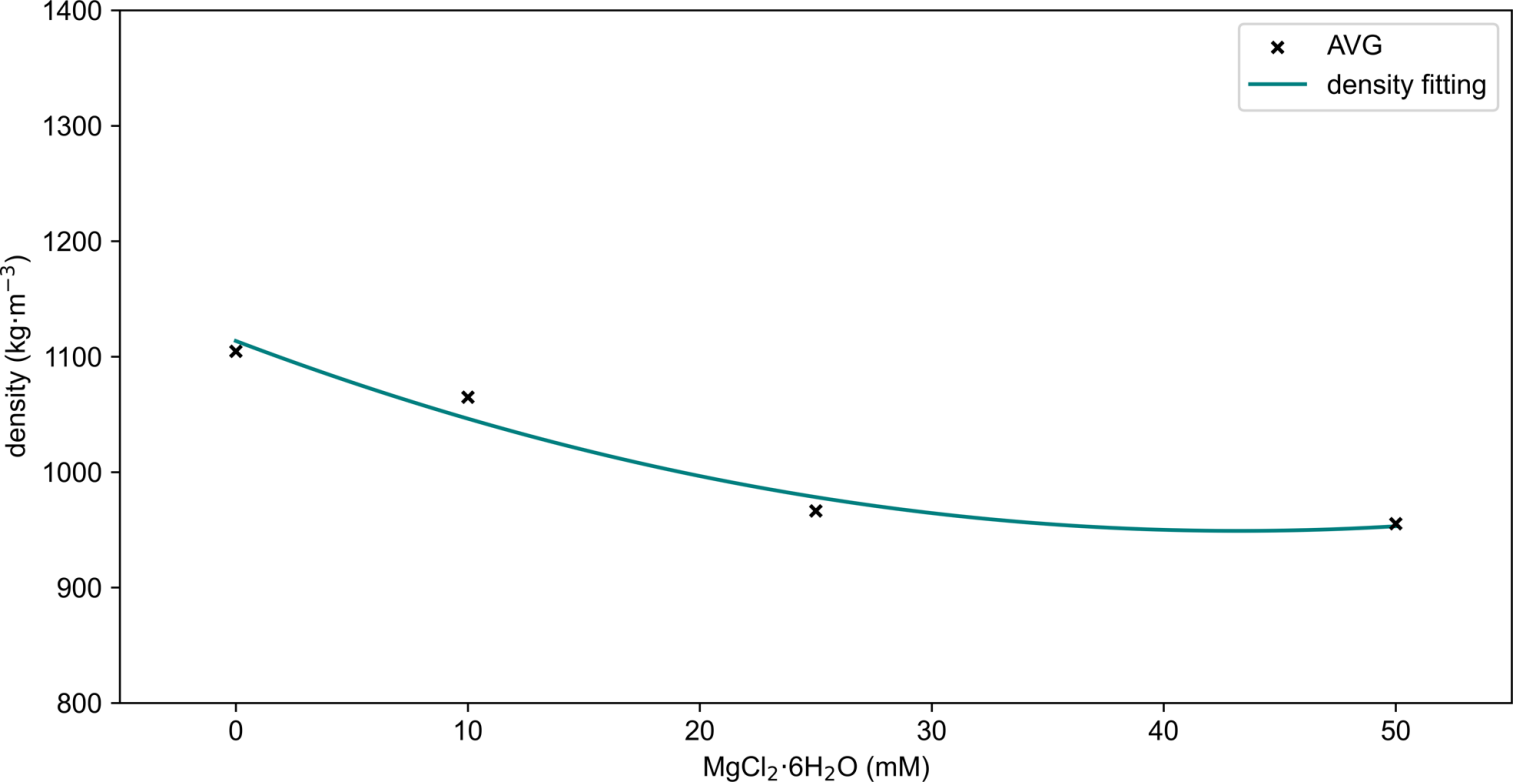
Mean density as a function of MgCl_2_·6H_2_O concentration. The *x*-axis represents the MgCl_2_·6H_2_O concentration in millimolar (mM), while the *y*-axis represents the density in arbitrary units. The black crosses (AVG) represent the mean density values for each concentration (0, 10, 20, 25, 30, 40 and 50 mM) based on experimental measurements. The teal solid line (density fitting) represents the equation simulation equation (2.2) of density as a function of MgCl_2_·6H_2_O concentration. This plot illustrates the relationship between MgCl_2_·6H_2_O concentration and the density of the ECM, highlighting the equation’s accuracy in predicting density within the range of 0 to 50 mM.


The results of the density measurements for the ECM samples (figure 2) were used as the basis for developing a quadratic regression equation (2.2) that describes the density of the ECM as a continuous function of MgCl_2_·6H_2_O concentration (*c*). Figure 3 presents the experimental data (discrete points) alongside the continuous quadratic regression curve for concentrations ranging from 0 to 50 mM. The coefficients *a*, *b* and *d* were determined empirically through quadratic regression, providing an equation that accurately fits the observed data.

(2.2) $$\begin{array}{c} \rho (c)=a\cdot c^{2}+b\cdot c+d\\ c\in R^{\geq 0},0\leq c\leq 50,a=0.088,b=-7.61,d=1113.59. \end{array}$$

### 2.3. Kinematic viscosity measurement and data extension approach

**Figure 4.**
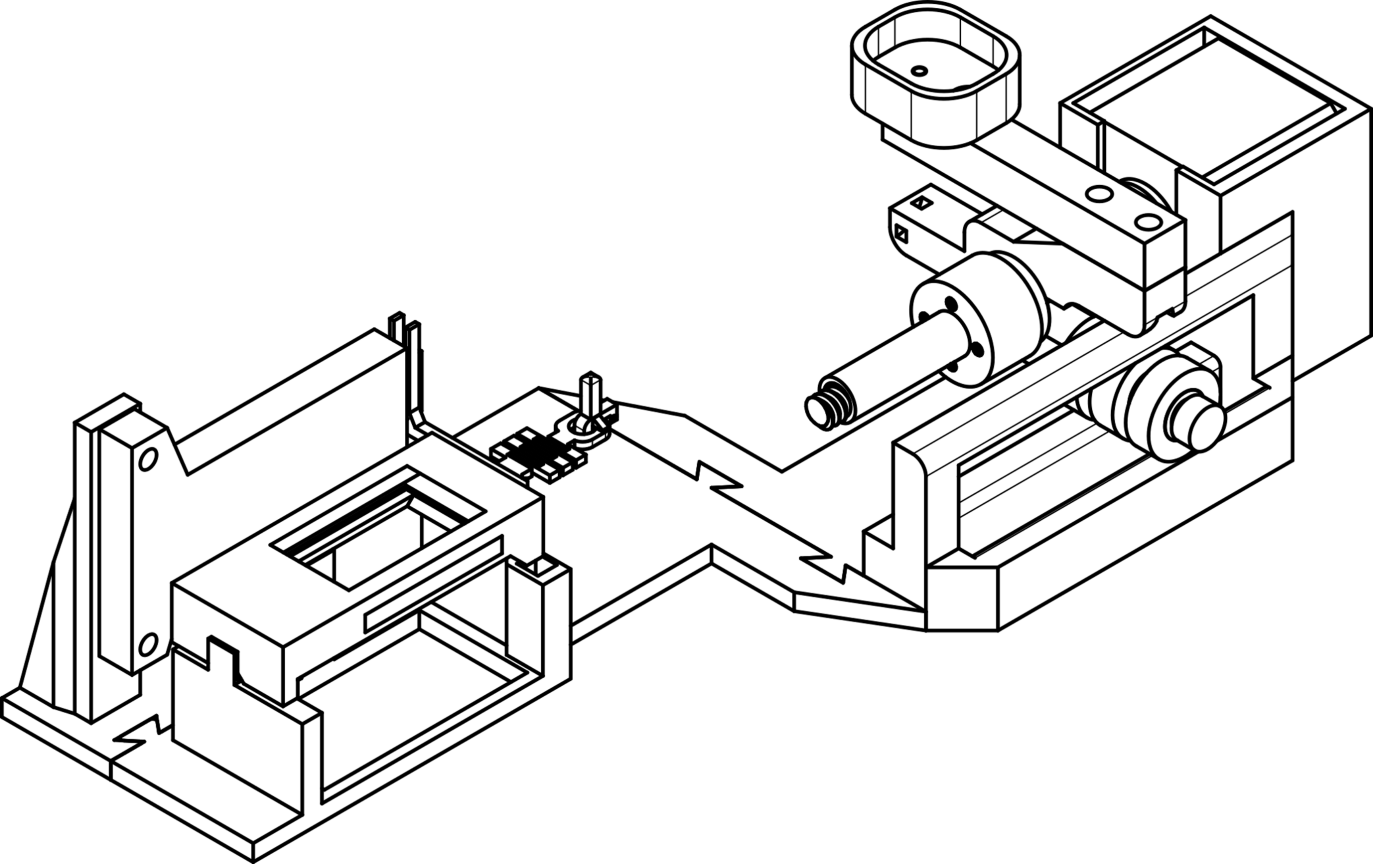
Technical illustration of the engineered viscometer for kinematic viscosity measurements. This viscometer is designed explicitly for accurate viscosity measurements in small biological samples. The device features a compact sample holder for milligram-scale sample sizes, allowing for precise control and minimal sample loss. The viscometer operates by measuring the flow resistance of the sample as it passes through the instrument’s channels, with the results used to determine the relative kinematic viscosity. The detailed operation, set-up, calibration and measurement protocols are provided in the supplementary material.

The sample preparation for kinematic viscosity measurement follows a similar procedure to that used for density measurement. Detailed technical specifications, including the operation, set-up, calibration and measurement protocols for the viscometer (figure 4), are provided in the supplementary material, where comprehensive instructions are available.

**Figure 5.**
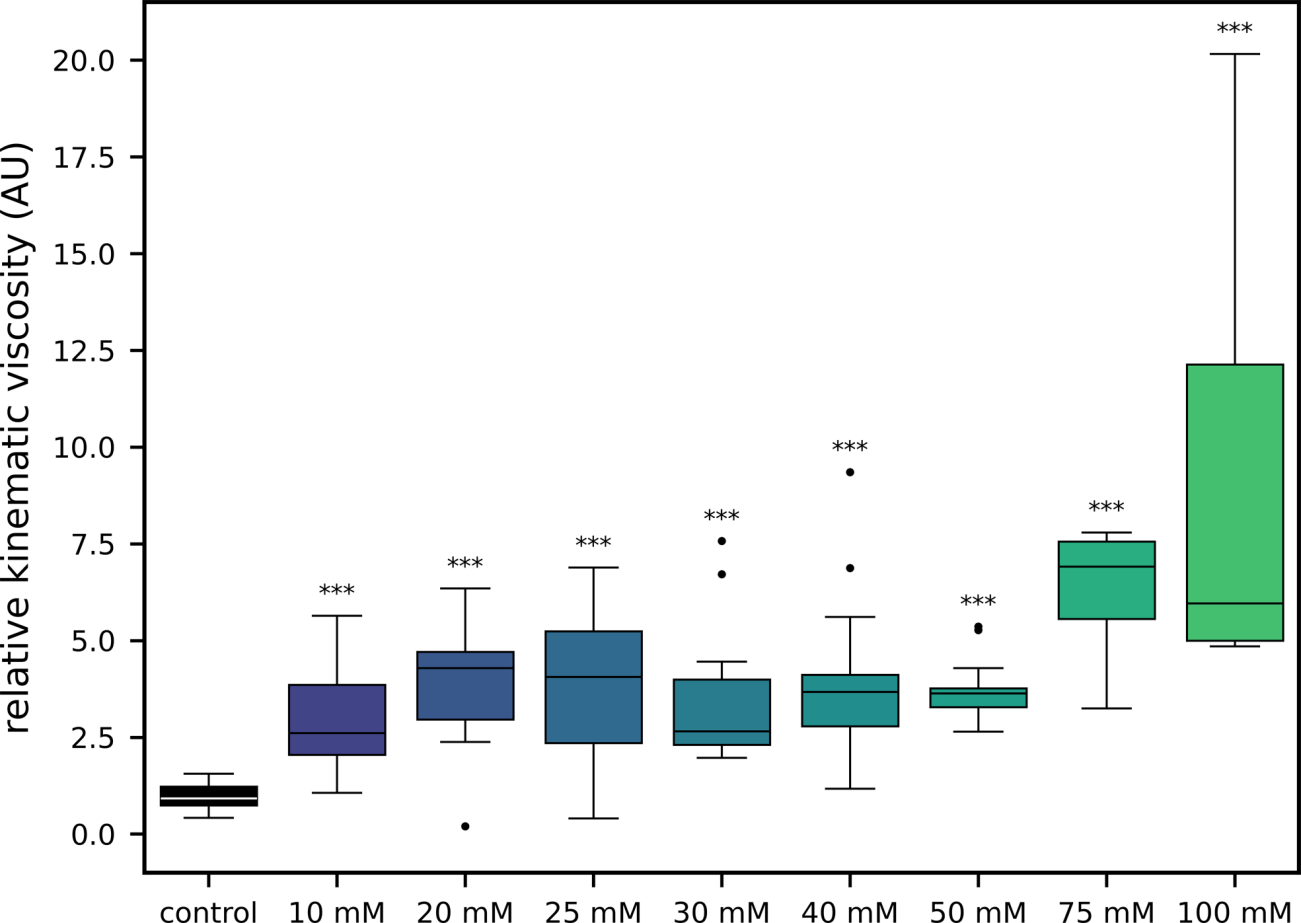
Box plot of relative kinematic viscosity of ECM samples across different concentrations of MgCl_2_·6H_2_O. The *y*-axis represents the relative kinematic viscosity in arbitrary units, while the *x*-axis represents the MgCl_2_·6H_2_O concentration in millimolar (mM). Only the ECM samples are visible in this plot. The control group (no MgCl_2_·6H_2_O) is shown on the far left, serving as the baseline for comparison. Each box represents the interquartile range (IQR), with the median indicated by the horizontal line inside the box. The whiskers extend to 1.5 times the IQR and the circles represent outliers. The data points are plotted for MgCl_2_·6H_2_O concentrations of 10, 20, 25, 30, 40, 50, 75 and 100 mM. The asterisks (***) above the boxes denote a statistically significant difference (*p* < 0.001) in relative kinematic viscosity compared with the control group. The plot illustrates that increasing concentrations of MgCl_2_·6H_2_O generally result in higher relative kinematic viscosity, with the most significant increase observed at 100 mM. This trend highlights the strong relationship between MgCl_2_·6H_2_O concentration and ECM viscosity, with potential implications for the organism’s network dynamics.

Using the data from figure 5, we calculated the mean relative kinematic viscosity for each measured concentration (0, 10, 20, 25, 30, 40 and 50 mM). We excluded the 75 and 100 mM concentrations as it was not possible to fully distinguish between the ECM sample and the organism at these higher concentrations. Excluding these higher concentrations ensures the accuracy and relevance of the equation.

**Figure 6.**
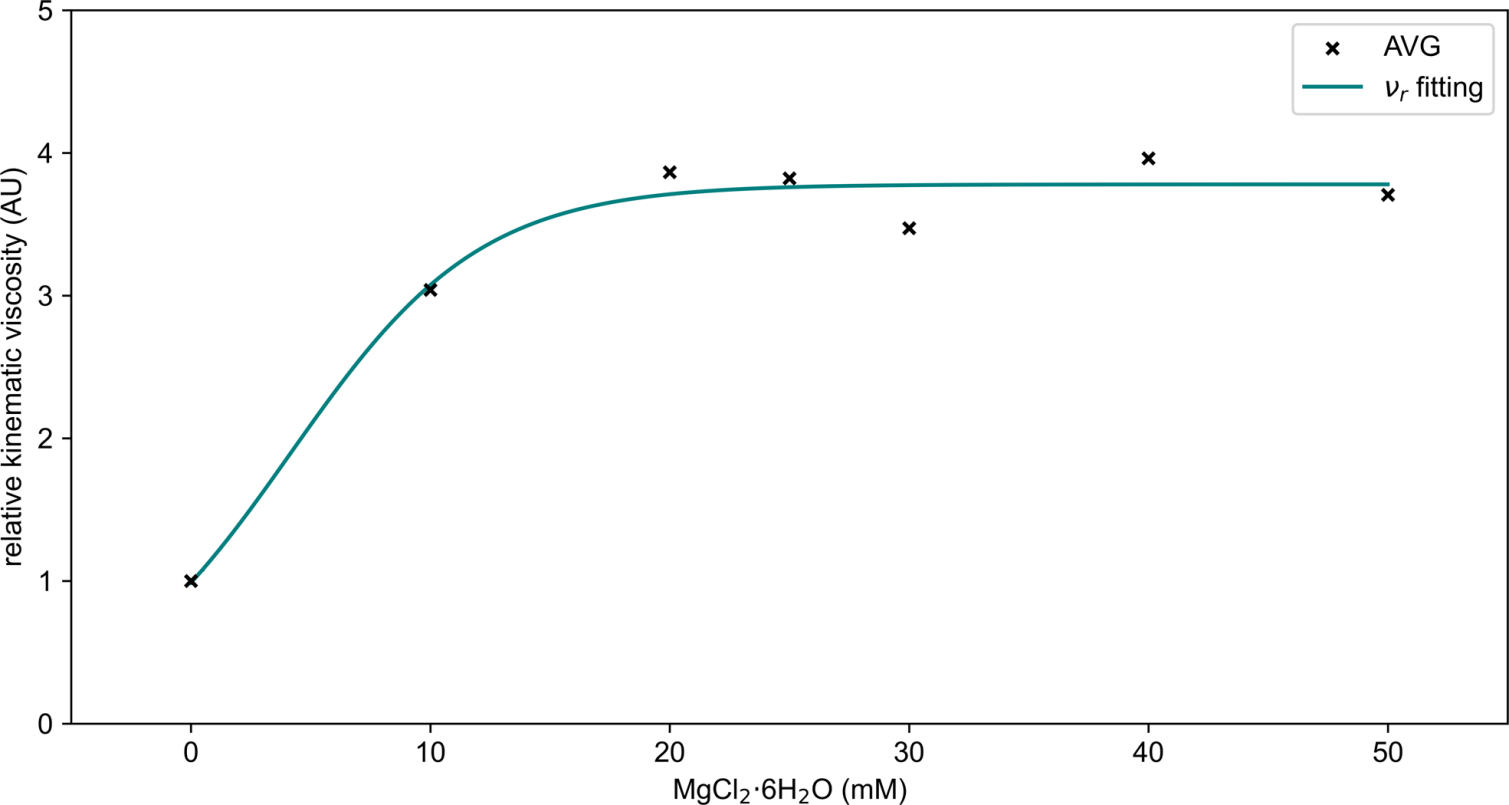
Mean relative kinematic viscosity (ν_r_) as a function of MgCl_2_·6H_2_O concentration. The *x*-axis represents the MgCl_2_·6H_2_O concentration in millimolar (mM), while the *y*-axis represents the relative kinematic viscosity in arbitrary units (AU). The black crosses (AVG) represent the mean relative kinematic viscosity values for each concentration (0, 10, 20, 25, 30, 40 and 50 mM) based on experimental measurements. The teal solid line (ν_r_ fitting) represents the equation simulation (equation 2.3) of relative kinematic viscosity as a function of MgCl_2_·6H_2_O concentration. The function excludes data from 75 and 100 mM concentrations due to difficulty distinguishing between the ECM sample and the organism at these higher concentrations. This plot illustrates the relationship between MgCl_2_·6H_2_O concentration and the relative kinematic viscosity of the ECM, highlighting the equation’s accuracy in predicting viscosity within the range of 0 to 50 mM.


The equation for relative kinematic viscosity of the ECM is presented in equation (2.3) and figure 6 provides a comparison of the calculated means with the equation simulation for concentrations ranging from 0 to 50 mM.

(2.3) $$\begin{array}{l} \nu_{r}\left(c\right)=\left(\frac{a\cdot c}{b+c}\right)\cdot e^{\alpha }+\frac{d}{a+e^{-f\cdot \left(c-g\right)}}\\ c\in R^{\geq 0},0\leq c\leq 50\\ a=0.032,b=-0.501,d=0.122,f=0.251,g=-9.541,\alpha =-7.838. \end{array}$$

To ensure that external factors, such as temperature and humidity did not influence the results, these parameters were closely monitored throughout the experiments. The average temperature was recorded at 24°C, with minimal variation between 23°C and 24.8°C and humidity levels remained stable around 55 *±* 9%. These consistent environmental conditions ensured that any changes in viscosity and network behaviour were primarily due to the manipulation of the ECM composition and not external fluctuations.

### 2.4. Experimental analysis of network growth and complexity in *Physarum polycephalum*

**Figure 7.**
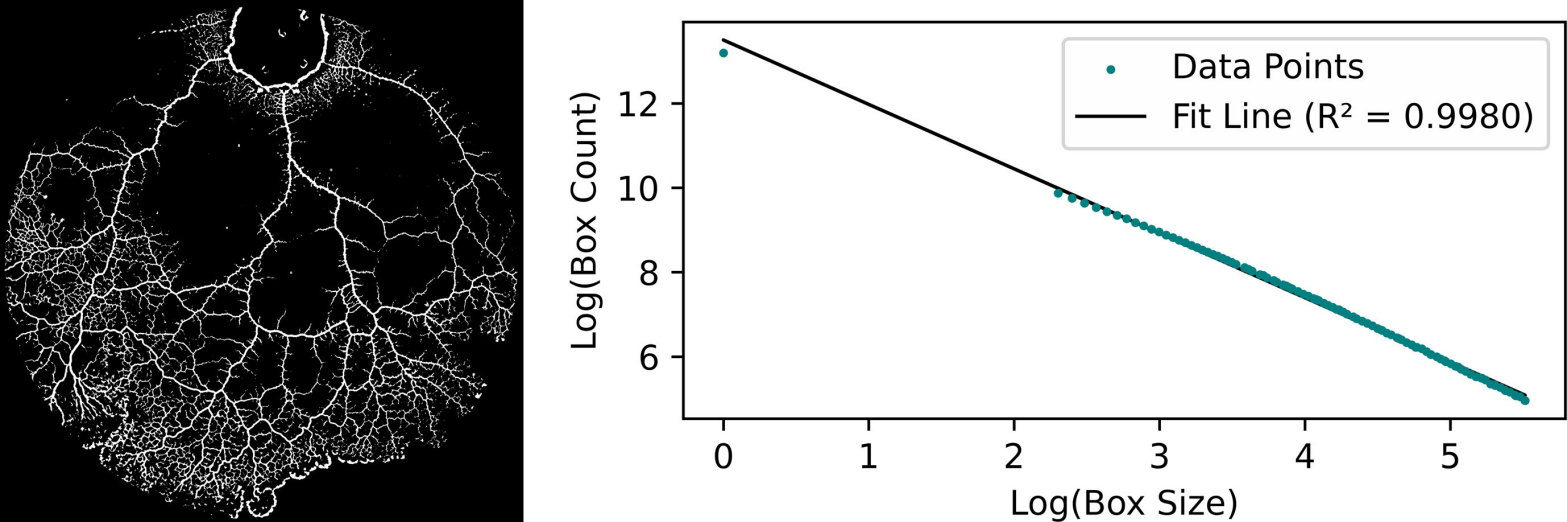
Illustration of the box-counting method for calculating FD. This figure calculates the FD of *P. polycephalum* networks. The left panel shows a preprocessed binary image of the slime mould, emphasizing its intricate vein-like structure. The right panel displays a log–log plot, where the *x*-axis represents the size of the boxes used to cover the network (on a logarithmic scale) and the *y*-axis represents the number of boxes needed to cover the network (also on a logarithmic scale). The slope of the fitted line in this plot provides the absolute value of the FD, offering a quantitative measure of the network's spatial complexity and self-similarity.

The analysis of network growth and complexity in *P. polycephalum* over 24 h was conducted through time-lapse imaging, capturing the organism’s expansion every minute. Using a Python algorithm, we measured the network size and calculated the FD of the network at each time point, utilizing the box-counting method. Figure 7 illustrates the box-counting method used in this study to calculate the FD. The left panel displays a preprocessed image of *P. polycephalum*, highlighting the organism’s network structure. The right panel presents a log–log plot where the *x*-axis represents the box size and the *y*-axis denotes the box count. The slope of the fitted line in the log–log plot corresponds to the FD’s absolute value, quantifying the network’s complexity and spatial organization.

This approach follows the same methodology outlined in a previous study, allowing for the consistent and accurate assessment of both network growth dynamics and structural complexity [37]. The study used two distinct approaches: a predictive model for network growth based on ECM viscosity and a descriptive equation for the network’s complexity, captured through FD analysis. The growth model predicts how the organism expands over time as a function of viscosity, while the FD equation describes the complexity of the network, independent of viscosity, as it transitions from adaptation to equilibrium.

#### 2.4.1. Model for predicting network size based on extracellular matrix viscosity

The network growth and size over time, represented by $A\left(t,\nu_{r}\right)$ in equation (2.4), is modelled as a function of time *t* (ranging from 0 to 24 h) and relative kinematic viscosity *ν_r_*. Equation (2.4) captures the dynamic evolution of network size through three key components: a scaling factor $\varphi \left(\nu_{r}\right)$, a power-law growth term $t^{\psi \left(\nu_{r}\right)}$ and an exponential decay term $\nu_{r}$. These components collectively describe the interplay between initial growth behaviour and long-term suppression influenced by viscosity. At early time points, the growth dynamics are predominantly governed by the power-law term, which reflects the system’s sensitivity to viscosity through $\psi \left(\nu_{r}\right)$. Over time, the exponential decay term, determined by $\omega \left(\nu_{r}\right)$, dominated, leading to a gradual stabilization of network size. This model effectively highlights the dual-phase behaviour of network development, with an initial rapid or slow growth phase followed by viscosity-dependent damping that curtails further expansion.


(2.4) $$\begin{array}{l} A\left(t,\nu_{r}\right)=\varphi \left(\nu_{r}\right)\cdot t^{\psi \left(\nu_{r}\right)}\cdot e^{-\omega \left(\nu_{r}\right)\cdot t^{2}}\\ t\in R^{\geq 0},0\leq t\leq 24,\nu_{r}\in R,0.9874\leq \nu_{r}\leq 3.7797. \end{array}$$

The function $\varphi \left(\nu_{r}\right)$ in equation (2.5) determines the scaling factor of the network size, modulating the overall magnitude of growth relative to viscosity. It is defined as a piecewise function to capture distinct behaviours at low and high viscosities. For low viscosities ($\nu_{r}$) < 3.75977, the scaling factor follows an exponential form combined with a power-law dependence. This structure implies that for small $\nu_{r}$, network size is exponentially suppressed, with sharp sensitivity to changes in viscosity. Conversely, for high viscosities $\nu_{r}\;>\;3.75977$, $\varphi \left(\nu_{r}\right)$ transitions to a fourth-degree polynomial. This form introduces smoother variations in network size as viscosity increases, reflecting a more complex relationship in environments with higher ECM viscosities. The dual structure of $\varphi \left(\nu_{r}\right)$ allows the model to accommodate both abrupt and gradual scaling behaviours depending on the viscosity range.


(2.5) $$\begin{array}{cl} \varphi \left(\nu_{r}\right)=e^{-a_{1}\cdot \nu_{r}}\cdot b_{1}\cdot \nu_{r}^{c_{1}} & \text{ if }\nu_{r}\leq 3.75977\\ \varphi \left(v_{r}\right)=a_{2}\cdot \nu_{r}^{4}+b_{2}\cdot \nu_{r}^{3}+c_{2}\cdot \nu_{r}^{2}+d_{2}\cdot \nu_{r}+e_{2} & \text{ if }\nu_{r}>3.75977. \end{array}$$

The term $\psi \left(\nu_{r}\right)$ in equation (2.6) governs the initial growth rate of the network by defining the power-law exponent in $t^{\psi \left(\nu_{r}\right)}$. Like $\varphi \left(\nu_{r}\right)$, $\psi \left(\nu_{r}\right)$ is piecewise-defined to reflect varying growth behaviours at different viscosity ranges. For low viscosities, $\psi \left(\nu_{r}\right)$ includes an exponential component combined with a polynomial term. This structure indicates that initial growth is highly sensitive to changes in viscosity, with the combined contributions of exponential and polynomial components shaping the early expansion of the network. At higher viscosities, the behaviour transitions to a quadric form modified by an exponential decay factor. This ensures that the growth rate decreases more gradually as viscosity increases, capturing the diminishing sensitivity of network expansion to viscosity in highly viscous environments.


(2.6) $$\begin{array}{cc} \;\;\;\psi \left(\nu_{r}\right)=a_{1}\cdot e^{b_{1}\cdot \nu_{r}}+c_{1}\cdot \nu_{r}^{d_{1}}\;\;\;\;\; & if\;\nu_{r}\leq 3.75977\\ \psi \left(\nu_{r}\right)=\left(a_{2}\cdot \nu_{r}^{2}+b_{2}\cdot \nu_{r}+c_{2}\right)\cdot e^{-d_{2}\cdot \nu_{r}}\;\;\; & if\;\nu_{r}>3.75977. \end{array}$$

The function $\omega \left(\nu_{r}\right)$ in equation (2.7) controls the rate of exponential decay in network growth, dictating how quickly the growth slows down over time. For low viscosities, $\omega \left(\nu_{r}\right)$ exhibits a quadratic dependence on viscosity, further amplified by an exponential factor. This structure models a rapid increase in damping with small changes in viscosity, leading to a swift stabilization of network size in low-viscosity environments. For high viscosities, $\omega \left(\nu_{r}\right)$ simplifies to a purely quadratic form, which captures smoother and more predictable decay behaviours. This simplification reflects the reduced variability in damping as the network grows under conditions of high viscosity, providing a consistent representation of low-term suppression.


(2.7) $$\begin{array}{cc} \omega \left(\nu_{r}\right)=\left(a_{1}\cdot \nu_{r}^{2}+b_{1}\cdot \nu_{r}+c_{1}\right)\cdot e^{\nu_{r}}\; & if\;\nu_{r}\leq 3.75977\\ \;\;\;\omega \left(\nu_{r}\right)=a_{2}\cdot \nu_{r}^{2}+b_{2}\cdot \nu_{r}+c_{2}\;\;\; & if\;\nu_{r}>3.75977. \end{array}$$

The overall model integrates these three components $\varphi \left(\nu_{r}\right)$, $\psi \left(\nu_{r}\right)$ and $\omega \left(\nu_{r}\right)$ to describe the complex interplay between viscosity and network growth dynamics. The scaling factor $\varphi \left(\nu_{r}\right)$ determines the baseline growth magnitude, reflecting the organism’s capacity to adapt to changes in viscosity. The power-law exponent $\psi \left(\nu_{r}\right)$ governs the rate of early expansion, highlighting how viscosity affects the organism’s initial adaptation strategies. Finally, the decay term $\omega \left(\nu_{r}\right)$ models the long-term suppression of growth, emphasizing the impact of viscosity on the stabilization phase. By using piecewise functions, the model captures both abrupt and gradual transitions in growth behaviour, making it robust across a wide range of viscosities.

We used a curve-fitting method to determine the model equation coefficients accurately. This approach involved fitting the model to the data for concentrations 0, 25 and 50 mM MgCl_2_·6H_2_O while intentionally excluding the 10 mM concentration data from the fitting process to evaluate the model’s predictive power later. The fitting process was optimized by minimizing the mean squared error (m.s.e.) between the model predictions and the experimental data. The resulting fitting precision, expressed as the m.s.e. in percentage, was 0.0229% for 0, 0.0762% for 25 and 0.0506% for 50 mM concentrations, demonstrating strong agreement between the model and the fitted data. We prepared a table (table 1) that contains the absolute values of the variables for the subequations *φ*(*ν_r_*), *ψ*(*ν_r_*) and *ω*(*ν_r_*).

**Table 1.** Coefficients used in the model for predicting network size based on ECM viscosity.

	*φ*(*ν_r_*)	*ψ*(*ν_r_*)	*ω*(*ν_r_*)
*a*_1_	0.853	0.01509	3.16 × 10^−5^
*b*_1_	6.613	1.0726	−0.0002022
*c*_1_	0.389	1.73876	0.0003956
*d*_1_	—	0.06608	—
*a*_2_	88822.584	−2.81788	−2.5116402
*b*_2_	−670160.693	21.00974	18.786904
*c*_2_	20096.232	−39.14962	−35.1266182
*d*_2_	9386038.401	−1.52188	—
*e_2_*	−17704756.283	—	—

Additionally, we processed an electronic supplementary material, video file to further explore and visualize the model’s behaviour across a broader range of viscosities. This video illustrates the model’s predictions at the experimental viscosities (0, 10, 25 and 50 mM MgCl_2_·6H_2_O) and for intermediate viscosities. To generate this visualization, we performed 600 tests between each experimental concentration range (0–10, 10–25 and 25–50), resulting in a slightly different resolution for each segment. Despite this variation, the movie effectively demonstrates the model’s ability to capture network growth dynamics across a continuous spectrum of viscosities, providing insight into the system’s behaviour at many interpolated points.

#### 2.4.2. Fractal dimension analysis of network complexity

FD is a robust measure to classify the network complexity of *P. polycephalum*. It captures the degree to which the organism’s network fills the available space, making it an ideal metric to quantify structural changes over time. In this study, we measured FD every minute for 24 h, analysing the network’s evolving complexity under different environmental conditions. Upon evaluating the data, we identified three distinct stages in the FD progression: the initial noise phase (0–2.5 h), the adaptation phase (2.5–15.5 h) and the equilibrium state (15.5–24 h). The initial growth phase exhibited excessive noise due to the rapid, unstable reorganization of the network, and as a result, we excluded this phase from further analysis. The adaptation phase reflects changes in network complexity caused by variations in viscosity and environmental conditions, while the equilibrium state marks a point at which the network complexity stabilizes, independent of external factors. Given that the FD converges to a similar value (approx. 1.533) across all conditions during the equilibrium state, we describe the network complexity with a viscosity-independent exponential function equation (2.8).

(2.8) $$\begin{array}{l} C\left(t\right)=D_{f}-e^{-\lambda \cdot t}\\ t\in R^{\geq 2.5},2.5\leq t\leq 24\\ \lambda =0.2776,D_{f}=1.533. \end{array}$$

This equation describes the exponential increase in complexity during the adaptation phase and its subsequent stabilization in the equilibrium state. The parameters *D_f_* and *λ* were determined through curve fitting, using data from all conditions simultaneously. The exponential nature of the equation accurately captures the rapid increase in complexity during the adaptation phase, followed by a plateau as the network reaches its final, stable configuration.

### 2.5. Statistical analysis

Data were tested for normality using the Shapiro-Wilk test, implemented in a custom Python (v. 3.10) script using the scipy.stats library (v. 1.8.0). For normally distributed data, a Student’s *t*‐test was performed to assess the significance of differences between groups. The Mann–Whitney *U* test was used for data that did not meet the assumption of normality. Significance levels are indicated by asterisks in the results: * denotes *p*-values *≤* 0.05, ** denotes *p*-values *≤* 0.01 and *** denotes *p*-values *≤* 0.001. In order to validate the models or equations, we calculated the m.s.e. using the sklearn.metrics library (v. 1.4.0rc1). Additionally, the model’s accuracy variation was expressed as a percentage, calculated by dividing the m.s.e. by the square of the range (max–min) and multiplying by 100. This approach provides a clear metric for assessing the model’s performance across different conditions.

## 3. Results

### 3.1. Characterization of extracellular matrix properties: density and kinematic viscosity

The density measurements of the ECM sampled from *P. polycephalum* provided a foundational parameter for converting dynamic viscosity into kinematic viscosity. Figure 2 shows the density values for both the Cell-ECM composite and the ECM samples across the control and three standard MgCl_2_·6H_2_O concentrations (10, 25 and 50 mM). No significant differences in density were observed between the different conditions, suggesting that the addition of magnesium chloride did not significantly affect the overall density of the samples.

**Figure 8.**
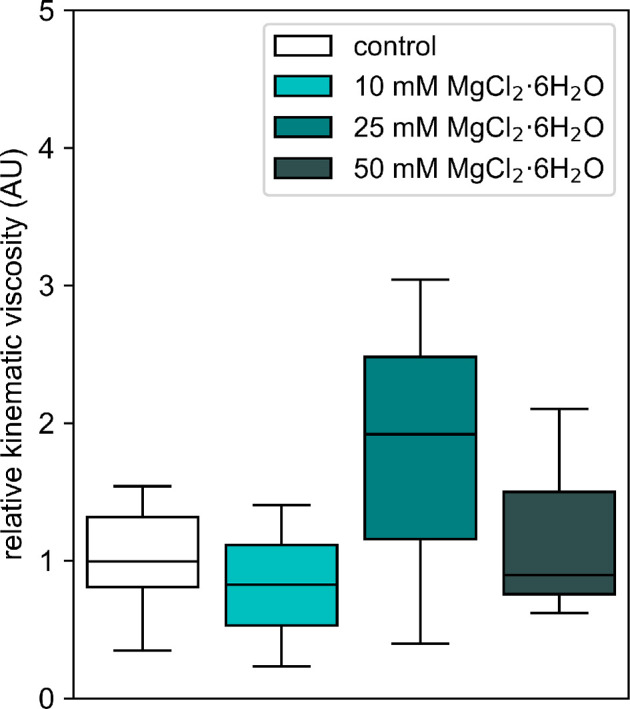
Box plot of relative kinematic viscosity of Cell+ECM samples across different concentrations of MgCl_2_·6H_2_O. The *y*-axis represents the relative kinematic viscosity in arbitrary units, while the *x*-axis represents the MgCl_2_·6H_2_O concentration in millimolar (mM). The control group (no MgCl_2_·6H_2_O) is shown on the far left, serving as the baseline for comparison. Each box represents the interquartile range (IQR), with the median indicated by the horizontal line inside the box. The whiskers extend to 1.5 times the IQR. The data points are plotted for MgCl_2_·6H_2_O concentrations of 10, 25 and 50 mM. No significant differences were found in the relative kinematic viscosity when comparing the different concentrations to the control group, indicating that for Cell+ECM samples, MgCl_2_·6H_2_O does not strongly affect viscosity at the tested levels.

The kinematic viscosity, derived from dynamic viscosity using the density measurements, is a critical parameter for understanding how the ECM viscosity influences the growth and structural complexity of the network in *P. polycephalum*. Two figures (figures 5 and 8) were prepared to present the kinematic viscosity data for the Cell-ECM and ECM samples, respectively. Figure 8 shows the kinematic viscosity of the Cell-ECM samples across the control and three magnesium chloride concentrations. The results indicate no significant differences in kinematic viscosity among these conditions. This lack of significance could be attributed to the ECM being just a minor component of the overall sample, with the organism constituting the central part, which may stabilize the Cell-ECM sample matrix, leading to no observable change in viscosity with the addition of magnesium chloride.

In contrast, figure 5 focuses on the ECM samples, where the results reveal significant differences in kinematic viscosity between the control and each of the magnesium chloride conditions. This finding is critical as it demonstrates that the engineered viscometer effectively captures the ECM viscosity variations arising from the different magnesium chloride concentrations. The significant differences in viscosity show the sensitivity of the system, which correlates with the changes in network growth and complexity. To report the kinematic viscosity as relative values rather than absolute ones stems from the specific constraints of the study. While effective for comparison, the self-engineered viscometer has yet to be calibrated against commercially available viscometers, which typically require larger sample sizes that are not feasible with the ECM samples. Given this limitation, reporting relative kinematic viscosity allows for accurate comparisons between conditions without the potential inaccuracies that might arise from absolute measurements. This approach ensures robust analyses and valid conclusions about the influence of viscosity on network dynamics.

To extend our analysis beyond the discrete magnesium chloride conditions initially tested, we developed equations to predict the density and relative kinematic viscosity as continuous functions of magnesium chloride concentration, denoted by *c*. This approach allows for a more flexible and comprehensive understanding of how varying magnesium chloride concentrations influence these fundamental physical properties across a broader range. The density (*ρ*) as a function of magnesium chloride concentration is described by the quadratic equation shown in equation (2.2), where the coefficients *a*, *b* and *d* were determined through curve fitting.

The equation’s accuracy is demonstrated in figure 3, where the teal solid line (density fitting) closely aligns with the experimental mean density values (black crosses) across all concentrations tested. This result shows that the equation provides reliable predictions of density changes as a continuous function of magnesium chloride concentration, with minimal deviations from the experimental data.

Similarly, the relative kinematic viscosity (*ν_r_*) is designed as a continuous function of magnesium chloride concentration using equation (2.3). As shown in figure 6, the equation captures the evolution of relative kinematic viscosity across the concentration range from 0 to 50 mM. The strong agreement between the equation and the experimental data highlights the accuracy of the fitting, with m.s.e. remaining below 0.3% across all conditions. By using continuous functions, we extend the analysis beyond the limitations of discrete data points, allowing for more precise predictions across a range of magnesium chloride concentrations. These equations enhance our understanding of how both density and kinematic viscosity are influenced by ECM composition and contribute to a deeper understanding of the physical parameters governing *P. polycephalum* network dynamics.

### 3.2. Network growth and network size

**Figure 9.**
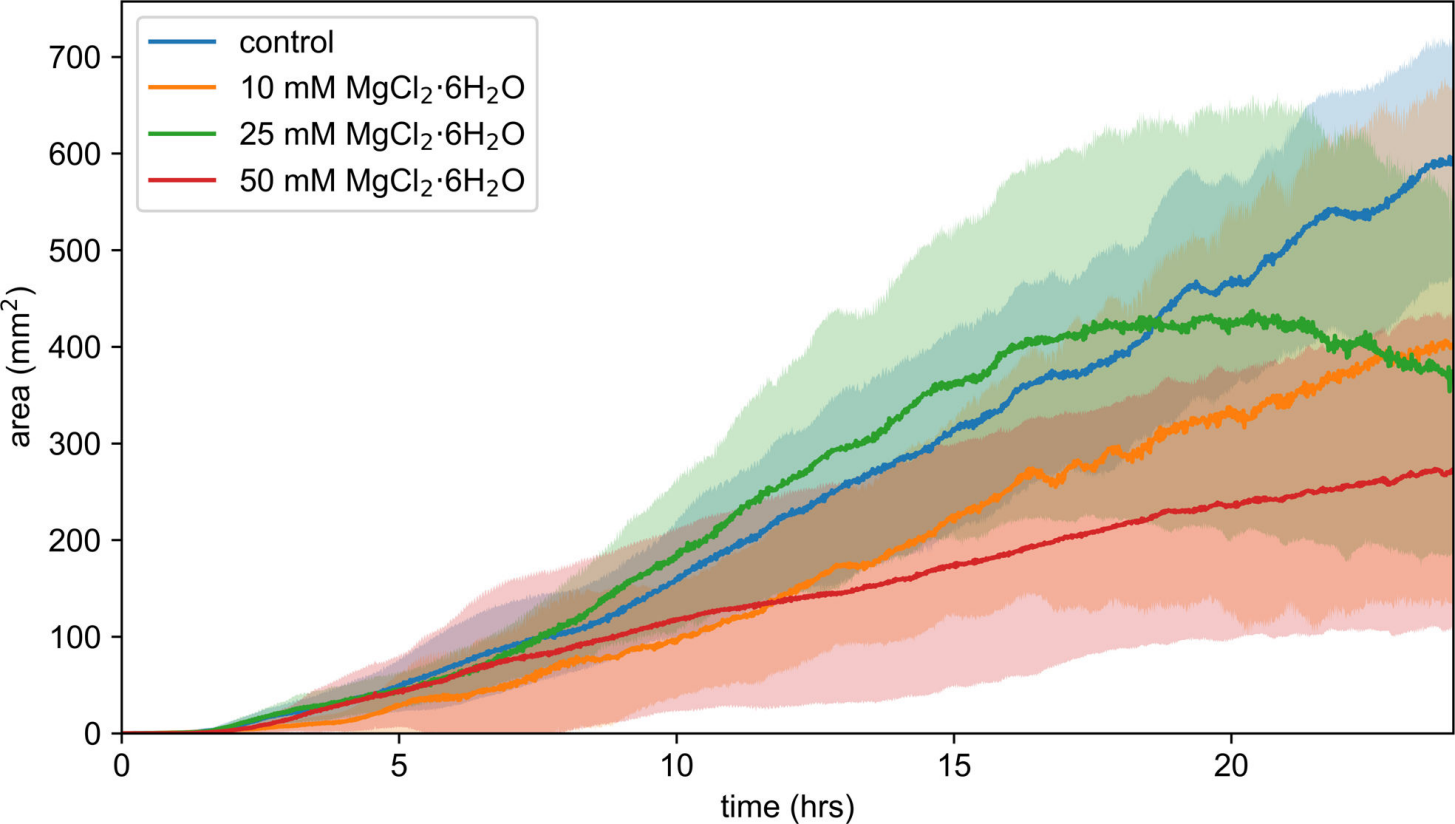
Area plot of *P. polycephalum* network growth over 24 h under different MgCl_2_·6H_2_O concentrations. The *y*-axis represents the network area in mm^2^, while the *x*-axis represents time in hours. The plot shows the growth of *P. polycephalum* networks for the control group (no MgCl_2_·6H_2_O, *n* = 15) and samples with 10 mM (*n* = 5), 25 mM (*n* = 7) and 50 mM MgCl_2_·6H_2_O (*n* = 7) concentrations. The different colours represent different MgCl_2_·6H_2_O concentrations. Each coloured area represents the range of network growth observed over multiple replicates, with the central line indicating the mean growth for each condition. The plot illustrates how different concentrations of MgCl_2_·6H_2_O affect the growth rate and overall network expansion of *P. polycephalum*. The control group shows the most significant growth, while higher concentrations of MgCl_2_·6H_2_O generally result in reduced growth rates and network expansion.

The network growth of *P. polycephalum* was tracked over 24 h, revealing distinct growth trajectories under different magnesium chloride concentrations. Figure 9 illustrates the network size as a function of time, showing that while all conditions led to network expansion, the rate and extent of growth varied significantly depending on the concentration of magnesium chloride. The plot demonstrates clear differences between conditions, with higher magnesium chloride concentrations generally resulting in slower network growth. Despite the variability within each group, the overall trend shows that increasing magnesium chloride concentration impedes the growth rate of the network.

**Figure 10.**
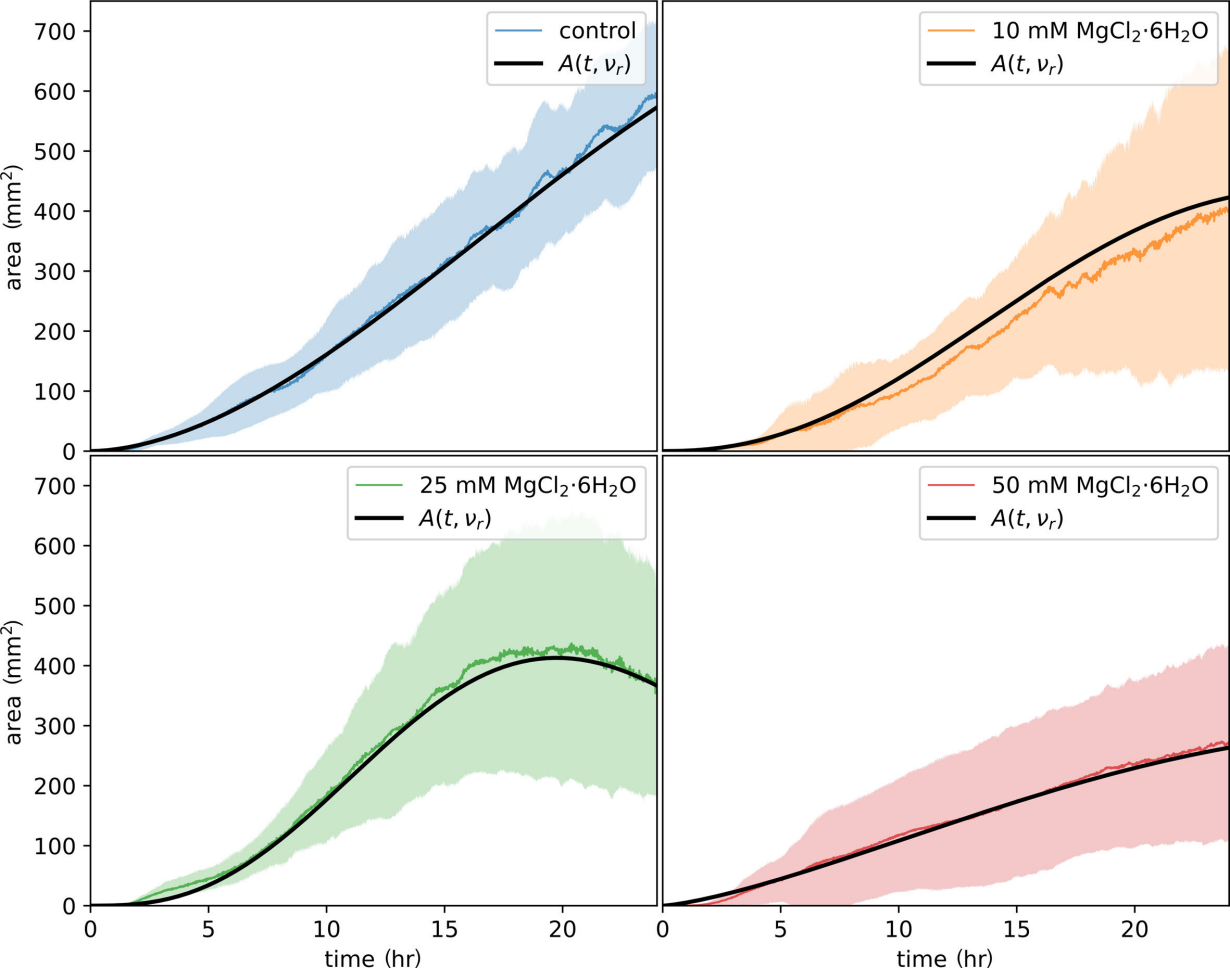
Model fitting and predictive performance of network growth at different viscosity conditions. This figure demonstrates the performance of the network growth model *A*(*t*, *ν_r_*) (black lines) in fitting and predicting the network size over time across different concentrations of MgCl_2_·6H_2_O, which affects the relative kinematic viscosity (*ν_r_*) of the ECM. The shaded regions in each plot represent the experimental data for the respective concentration, while the black lines indicate the model’s performance. For the control (0 mM, blue), 25 mM (green) and 50 mM (red) conditions, the black curves represent the fitting results, as these concentrations were included in the model fitting process. The model shows strong agreement with the experimental data, as evidenced by the close alignment of the black curves with the shaded regions, demonstrating the model’s ability to capture network growth behaviour with high precision. In contrast, the 10 mM (orange) plot demonstrates the model’s predictive power. The black curve represents the model’s prediction for this condition since the 10 mM concentration was not included in the fitting process. The close alignment of black curves with experimental data demonstrates the model’s accuracy and ability to predict network growth under varying viscosity conditions, underscoring the impact of ECM viscosity on network expansion.

A mathematical model (equation (2.4)) was developed to describe and predict the network growth behaviour. The solid lines in figure 10 represent the model’s performance, with fitting results for the control, 25 and 50 mM conditions and a prediction for the 10 mM condition. The close alignment between the model and the experimental data for the fitted concentrations and the accurate prediction for the 10 mM condition demonstrates the model’s robustness in capturing the growth dynamics of *P. polycephalum* under varying viscosity conditions.

The model’s predictive power for the 10 mM condition was further quantified using the m.s.e., which measures how well the model’s prediction aligns with the experimental data. For the 10 mM concentration, the m.s.e. was calculated as 0.36%, indicating that while the model was not explicitly fitted to this condition, it still demonstrated high predictive accuracy. This result further validates the model’s ability to generalize across different viscosity conditions, even when specific data points are not included in the fitting process.

These findings highlight the critical influence of magnesium chloride concentration on the network expansion of *P. polycephalum*, with higher concentrations limiting growth while maintaining accurate predictions through the model. This close agreement between the model and experimental data underlines the model’s reliability in predicting network growth under varying ECM conditions.

### 3.3. Fractal dimension analysis

**Figure 11.**
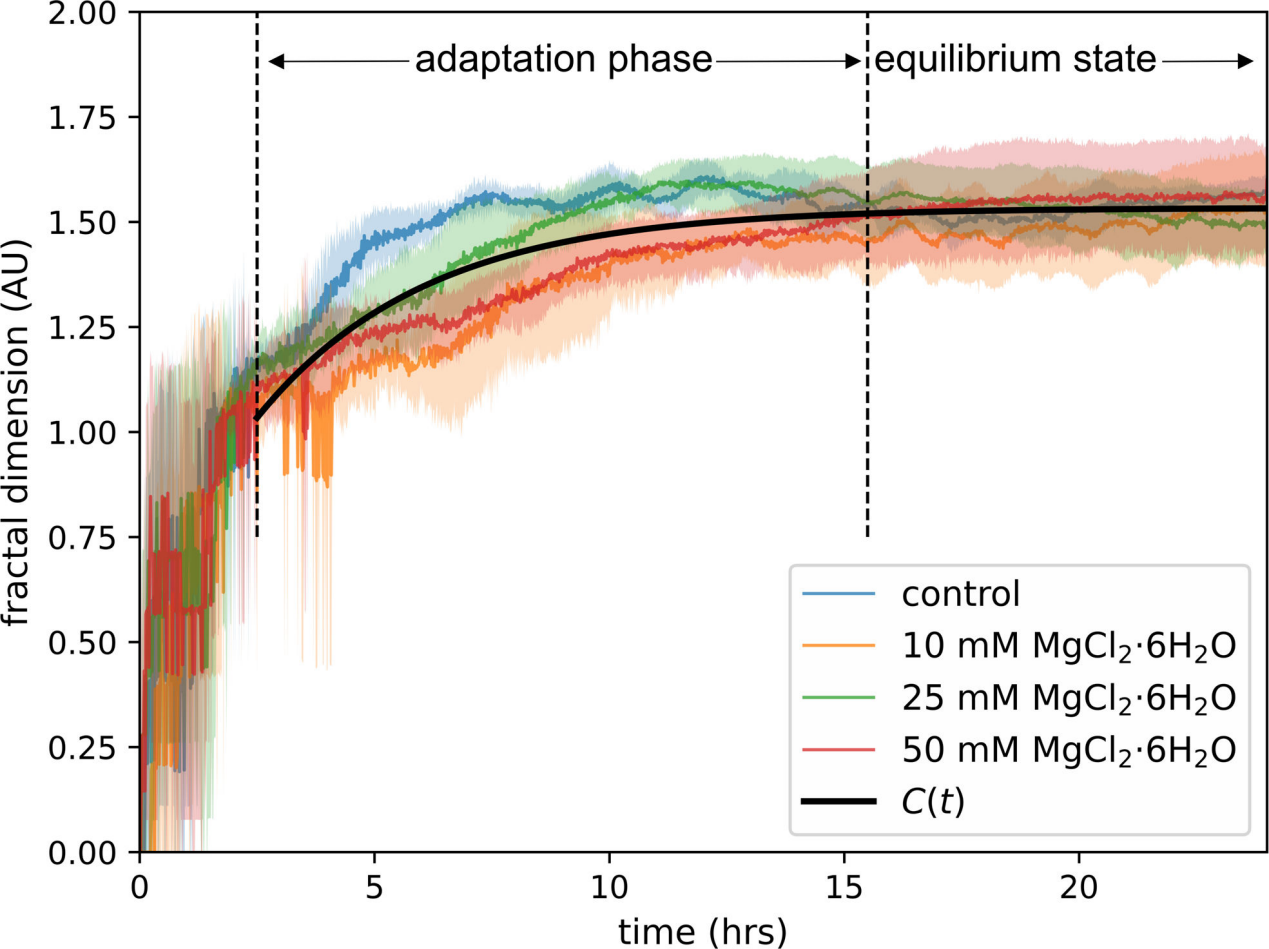
FD analysis of *P. polycephalum* network complexity over time. This figure shows the FD of *P. polycephalum* networks over time under different magnesium chloride (MgCl_2_·6H_2_O) concentrations: control (blue, *n* = 5), 10 (orange, *n* = 5), 25 (green, *n* = 7) and 50 mM (red, *n* = 7). The black solid line represents equation (2.8), capturing the general trend of network complexity over time. The experimental data for all conditions converges to a similar FD (approx. 1.533) during the equilibrium stage.

The FD analysis provides a detailed view of the structural complexity of *P. polycephalum* networks over time, particularly under varying magnesium chloride concentrations. Figure 11 illustrates how the FD evolves from 0 to 24 h, offering insight into how the network’s complexity changes as the organism expands and stabilizes its structure. The FD evolution exhibits distinct phases, highlighting different stages of network development. The FD is highly variable during the initial growth phase (0−2.5 h), with significant perturbations and noise. This phase is followed by the adaptation phase (approx. 2.5−15.5 h), where the FD increases steadily, showing the organism’s response to different magnesium chloride concentrations and environmental factors. Finally, the FD reaches the equilibrium state (after approx. 15.5 h), where all conditions converge to a similar FD of approximately 1.533, indicating a stable and fully developed network structure across all conditions.

To further describe the FD behaviour, we fitted an exponential equation (2.8) to capture the general trend of network complexity. This equation describes the rapid increase in complexity during the adaptation phase, which eventually reaches a plateau in the equilibrium state. The fit was performed across all conditions simultaneously and the goodness-of-fit was assessed using the m.s.e. for each condition: control—3.33%, 10 mM—1.59%, 25 mM—1.25% and 50 mM—0.71%. The low m.s.e. values across all conditions demonstrate the robustness and accuracy of the exponential equation in describing the FD behaviour despite the differences observed during the adaptation phase.

## 4. Discussion

This study explored the influence of ECM viscosity on the network growth and structure of *P. polycephalum*, revealing that variations in viscosity significantly impact the organism’s behaviour and structural organization. The network expansion rate was inversely correlated with viscosity, confirming that higher viscosity creates greater shear forces, which slow down cytoplasmic streaming and reduce the organism’s ability to extend its protoplasmic veins. This observation supports previous research showing that viscosity and shear forces play a crucial role in patterning tube structures in *P. polycephalum* networks [24,25,38].

The growth model developed in this study effectively captures the influence of ECM viscosity on the network expansion of *P. polycephalum* through a combination of critical mathematical components. The model (equation (2.4)) integrates a power-law growth term and an exponential decay term, reflecting the dynamic interplay between the organism’s initial expansion and the eventual suppression of growth over time. The power-law term captures the early-phase behaviour, where the organism’s growth accelerates or decelerates depending on the viscosity. At the same time, the exponential decay represents the long-term limitations imposed by increasing viscosity. This power law and exponential decay combination is particularly suited for biological systems where growth is initially rapid but subject to physical constraints over time. The model’s high-fitting accuracy for 0, 25 and 50 mM concentrations and its predictive power for the 10 mM concentration demonstrate its robustness. The ability of the model to generalize and predict growth across different conditions suggests its potential for broader application in understanding the growth dynamics of similar biological systems under varying physical constraints. The combination of power-law growth and exponential decay terms may also be applicable to other systems that exhibit rapid early growth followed by constraint-limited expansion, including tissue growth, bacterial colony formation or biofilm development.

One of the most intriguing findings of this study is the identification of three distinct phases in the development of the FD of *P. polycephalum*, which is effectively captured by the FD function (equation (2.8)). During the first 2.5 h of growth, the network undergoes rapid and unstable reorganization, leading to high variability and noise in the FD measurements. This initial growth phase reflects the organism’s early attempts to establish its network, but it was excluded from further analysis due to the high noise level. Next is the adaptation phase, where the slime mould adjusts its internal architecture in response to external conditions, particularly ECM viscosity. The FD equation captures this dynamic shift as the network complexity increases in response to the viscosity and other external factors. At approximately 15.5 h, the network reaches the equilibrium state, where the FD stabilizes and converges to a consistent value across all viscosity conditions. Remarkably, this convergence to a uniform FD value during the equilibrium state indicates that regardless of the differences in growth rate caused by varying viscosities, *P. polycephalum* ultimately reaches a comparable level of network complexity. This suggests that the organism optimizes its network structure for efficiency and robustness in a viscosity-independent manner once it has adapted to its environment. Previous research has already demonstrated *P. polycephalum*’s remarkable ability to adjust its network in response to environmental challenges, optimizing resource distribution and maintaining resilience under various conditions [2,20,39,40]. The ability of the slime mould to stabilize its complexity under different conditions points to an evolutionary strategy to optimize resource distribution and resilience, where network efficiency and robustness are balanced against the energy invested in maintaining the network. This FD equation provides a valuable framework for understanding how biological systems achieve structural balance in response to environmental challenges, particularly regarding network architecture and resource optimization. The ability to adapt network complexity independent of environmental viscosity may also reflect general principles in biological systems, where structural efficiency and robustness are prioritized. This model could inform studies of other organisms or engineered systems, where environmental pressures shape network architecture.

While the models performed well in capturing network growth and complexity, they have limitations. One key limitation is that the models focus primarily on viscosity as the dominant factor influencing network dynamics. Other factors such as nutrient availability, ion concentrations or environmental stressors may also significantly shape network behaviour. However, these still need to be fully integrated into the current models. These additional variables could provide a more comprehensive understanding of *P. polycephalum*’s adaptive strategies. Future work should aim to expand these models by considering such factors, potentially leading to even more accurate predictions of network behaviour under varying environmental conditions.

The engineered viscometer demonstrated its ability to accurately measure ECM viscosity in samples as small as a few milligrams, overcoming the limitations of conventional viscometers, which often require significantly larger sample volumes. This capability is particularly crucial in studies involving limited biological material. Most commercially available viscometers are designed for larger sample sizes and are often specialized for low- or high-viscosity measurements. For example, the U-tube viscometer can handle small samples with low viscosity, or a Brookfield viscometer, which requires a sample size of a few hundred millilitres. This specialization limits their flexibility, particularly when handling a wide range of viscosities or when sample volume is constrained. In contrast, the engineered viscometer in this study is versatile in handling high-viscosity samples, although its performance with low-viscosity samples remains to be tested. However, a fundamental limitation of the viscometer is that its results must be normalized due to the need for more direct calibration with commercially available instruments. Without this calibration, the absolute viscosity values measured in this study should be interpreted cautiously, as they have not been benchmarked against standard devices. Further calibration against established viscometers, typically requiring larger samples, will be essential for providing definitive viscosity measurements. Despite these limitations, the viscometer shows considerable promise as a tool for biomechanical studies where precise viscosity measurements from minimal sample volumes are required. Future refinements, including performing measurements in a chamber with precise temperature and humidity control, would probably reduce environmental variability and improve accuracy. Although temperature and humidity were maintained within a controlled range (23–24.8°C and 55 ± 9%, respectively), tighter environmental regulation in future experiments could further reduce variability and enhance measurement reliability. For instance, performing viscosity measurements in a chamber with precise temperature and humidity controls would ensure greater repeatability and accuracy. Incorporating environmental factors, such as nutrient gradients or external stressors into the models could also enrich our understanding of how these variables interact with ECM viscosity to shape network dynamics.

Addressing these limitations could expand the viscometer’s applications, making it a valuable tool in a broader range of scientific and medical fields. Its ability to handle small sample sizes could be instrumental in tissue mechanics, where ECM viscosity plays a critical role in processes like tumour progression [41–43] and modern biological and medical applications, such as diagnostic tissue analysis, microfluidic systems and personalized medicine.

In conclusion, this study highlights the critical role of ECM viscosity in shaping the network growth and complexity of *P. polycephalum* and extends the relevance of these findings to broader biological systems. The mathematical models developed here offer a robust framework for predicting how physical properties, such as viscosity, influence network formation and structural complexity. These models can serve as a foundation for future research exploring adaptive network behaviours in other systems, including bioengineered networks or even computational models inspired by biological processes.

Although the viscometer requires further validation and refinement for application in other fields, its ability to measure viscosity in small sample volumes has already demonstrated significant potential in biomechanical research. With continued refinement of the device and integration of additional factors into the models, this research opens new pathways for understanding the complex interplay between physical properties and biological functions in adaptive networks. Overall, the combination of predictive modelling and advanced measurement techniques used in this study sets the stage for a deeper understanding of how physical constraints shape biological networks, with implications for both fundamental biology and practical applications in biomedical engineering.

## Data Availability

Data and Code are deposited in the Dryad repository at [44]. Electronic supplementary material is available online [45].
